# Adverse childhood experiences and subjective cognitive decline among transgender adults

**DOI:** 10.1002/alz.088507

**Published:** 2025-01-09

**Authors:** Jobina Chiow, Ethan C. Cicero

**Affiliations:** ^1^ Emory University, Atlanta, GA USA

## Abstract

**Background:**

Adverse childhood experiences (ACEs) are associated with an increased risk of developing chronic health conditions later in life, including Alzheimer’s disease and related dementias (ADRD) and subjective cognitive decline (SCD), a self‐reported confusion and memory loss and one of the first clinical manifestations of ADRD. While ACEs and SCD have both been individually studied in transgender and nonbinary (TGN) adults, no study has examined the relationship between ACEs and SCD among this population. This study sought to establish the prevalence of ACEs and their association with SCD among TGN adults.

**Method:**

2019‐2021 Behavioral Risk Factor Surveillance System (BRFSS) data, representing 16 U.S. states that assessed ACEs, SCD (confusion/memory loss happening more often/getting work over previous 12months), and self‐reported gender identity were used to determine the association between ACEs and SCD among TGN adults aged 45+ (*N* = 206). Pearson’s chi‐squared/Fisher’s exact tests assessed the association between ACEs (individual, categorical, sum score) and SCD. Crude and adjusted odds ratios (aOR) along with 95% confidence intervals (CI) were calculated to investigate the associations between ACEs (individual, categorical, sum score) and SCD.

**Result:**

18% (*n* = 38) of TGN adults in the sample reported SCD, 60% (*n* = 120) experienced any ACE, 20% (*n* = 41) 1 ACE, and 18% (*n* = 37) experienced ≥4 ACEs. Nearly 50% experienced childhood abuse (*n* = 94) or household dysfunction (*n* = 92); the most prevalent ACE was verbal abuse by parent/adult in the home. Among those endorsing SCD, 34% (*n* = 13) reported ≥4 ACEs, 73% (*n* = 26) childhood abuse or household dysfunction (*n* = 27). Most ACES were significantly associated with and increased the risk of SCD, even after adjusting for BRFSS year, age, race, education, and employment. The odds of SCD increased 40% as the number of ACEs increased (aOR = 1.4, 95% CI:1.2‐1.6, *p*<0.0001). The odds of SCD were significantly higher with childhood abuse (aOR = 4.3, 95% CI: 1.88‐10.02, *p*<0.01) or household dysfunction (aOR = 4.7, 95% CI: 2.00‐11.07, *p*<0.01).

**Conclusion:**

A lifecourse perspective is needed when examining the brain health of TGN adults. Childhood abuse and household dysfunction are risk factors for SCD. ACE screening/interventions for TGN youth are needed to help reduce the risk of SCD and cognitive impairment later in life.

